# New insights and opportunities from taking a biomechanical perspective on plant ecology

**DOI:** 10.1093/jxb/erac007

**Published:** 2022-02-24

**Authors:** Ulrike Bauer, Simon Poppinga

**Affiliations:** 1 School of Biological Sciences, University of Bristol, 24 Tyndall Avenue, Bristol BS8 1TQ, UK; 2 Botanical Garden, Technical University of Darmstadt, Department of Biology, Schnittspahnstraße 2, D-64287 Darmstadt, Germany

**Keywords:** Climate change, ecomechanics, field work, functional morphology, interdisciplinary, mechanical ecology, mechanical stress

**Mechanical ecology is an emerging interdisciplinary field at the intersection of biomechanics and field ecology. The development of ever smaller and more powerful portable devices for measurement and data acquisition has boosted the number of biomechanical field studies in recent years, shedding new light on often neglected, but crucial factors influencing mechanical aspects of plant ecology. This Special Issue showcases recent scientific and technological advances, and highlights the importance of considering the mechanical aspects of plant ecology, as well as putting biomechanical mechanisms into a biologically relevant real-world context.**

‘there is an underlying world with which life must contend’

Steven Vogel (1988)

All matter, including life on Earth, is subject to the rules of physics. Physical forces govern processes at all scales from atoms to ecosystems, and considering these forces is essential for a comprehensive understanding of plant ecology and evolution (Box 1). For example, the increasing frequency of severe weather events as a result of global climate change leads to ever higher mechanical stresses on plants and the soil that supports them (Ummenhofer and Meehl, 2017; Rädler *et al*., 2019), but how plants cope with such dramatic changes, which mainly occur in evolutionarily irrelevant time scales, is largely unknown (e.g. Onoda *et al*., 2010; Heredia-Guerrero *et al*., 2018; Johnson *et al*., 2019). Mitigating the immediate effects of these extreme weather events therefore requires an integrative understanding of the physical factors influencing natural vegetation and crops in order to understand plant responses to increasing mechanical stresses, but also the potential of plants to mitigate the effects of climate change-induced alterations of weather patterns. The unprecedented challenges of our time—climate change, habitat and biodiversity loss, food security for a growing world population, and accelerating loss of arable soils to erosion—require increasingly innovative and interdisciplinary approaches to solve (Glamann *et al*., 2017; Middendorf *et al*., 2020). This Special Issue explores one such interdisciplinary approach: the very promising and timely fusion of biomechanics and ecology (Box 1).

**Box 1.**

A multitude of physical forces act upon plants, and the resulting mechanical stresses have shaped both the overall growth forms and the composite cellular materials of extant plant species. Stems and branches must be strong enough to resist bending and buckling under the plant’s own load. Moreover, wind gusts and waves shock-load plants with extremely high forces that can cause catastrophic breakage or uprooting. The impact forces of raindrops and hailstones may appear insignificant in comparison; however, their effect is amplified by the concentration of force on a very small area, as is demonstrated by the erosive power of rain. Waves and tides both cause strong cyclic loading in opposite directions, while rain and hail impacts cause characteristic damped oscillations of the affected plant part.

However, mechanical forces not only pose the risk of tissue damage; they can also contain valuable information, and plants have evolved a multitude of mechanosensory structures and mechanisms to exploit this information source. A growing body of evidence shows that plants sense mechanical stresses in expanding cells walls and integrate this information with other factors such as morphogen gradients to establish polarity in developing tissues and coordinate cell divisions and organ differentiation. Microvibrations caused by the chewing mandibles of insects alert plants of herbivore attacks and kick-start biochemical defence pathways. Electrostatic forces not only function as signals for pollinators, but have also been shown to directly aid pollen transfer between flower and bee.

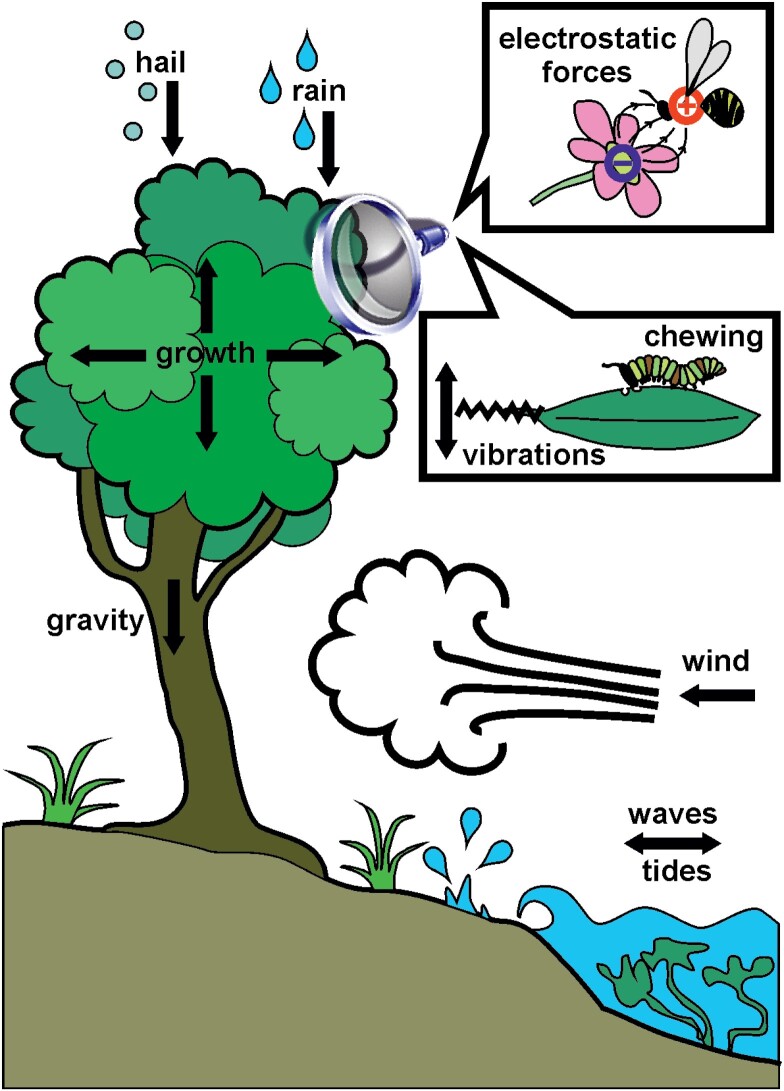

Biomechanics is an engineering–mathematics-based approach to understanding biological questions and processes (Vogel, 1988). It has its roots in zoological, biomedical, and sports science, but the impact and popularity of plant biomechanics is rapidly growing. This is evidenced by the emergence of several dedicated conferences (e.g. the International Plant Biomechanics Conference), and by multiple special issues in journals, including two in JXB over the past decade (in 2019, Plant Biomechanics: Force, Form, and Function; and in 2013, Plant Biomechanics and Mechanobiology). Plant ecology, however, is traditionally looking towards chemistry and physiology to explain responses of plants to biotic and abiotic stress factors, and climate change research focuses largely on the effects of rising temperature, increasing frequency and severity of droughts, elevated CO_2_ availability, and decreasing soil fertility on natural vegetation and agricultural crops (Pareek *et al*., 2020). Mechanical aspects of plant ecology thus remain poorly studied to date, and plant biomechanics is still largely confined to the lab; however, the key to fully understanding the impact of mechanical factors is to research them in the field, under natural conditions (Bauer *et al*., 2020; Niklas and Telewski, 2022).

From the obvious (e.g. wind, waves, and impacts from rain and hail) to the more obscure (e.g. gravitational and electrostatic forces, and microvibrations), mechanical factors affect virtually every aspect of plant life (Read and Stokes, 2006). In this issue, Niklas and Telewski (2022) explore how mechanical constraints and trade-offs played a key role in shaping modern plants as they transitioned from marine to terrestrial environments and colonized the vertical growth space, competing for light. They show how plant material properties and growth responses to mechanical loading evolved over time to allow them to grow taller and resist increasing mechanical loads; however, increasing height comes with additional challenges for water transport which may conflict with the demands for structural reinforcement (Baer *et al*., 2021). Lianas, epiphytes, and epi-parasites circumvent this conflict by largely foregoing structural reinforcement and instead relying on other plants for vertical support; here, the crucial mechanical adaptations are for secure attachment to the host tree, as shown for passionflowers and mistletoes in this issue (Klimm *et al*., 2022; Mylo *et al*., 2022).

Attachment (or lack thereof) is also crucial for plant–insect interactions such as plant carnivory, herbivory, and pollination (Whitney and Federle, 2013). Mechanical factors control the timing of pollen release (Nelson *et al*., 2012; Timerman and Barrett, 2021; Vallejo-Marin, 2022) and, very recently, the act of pollination itself has been shown to rely on attractive forces between flowers and bees with opposing electrostatic charges—prompting the launch of the subfield of electrical ecology (England and Robert, 2021). Electrostatic charges not only promote the movement of pollen between flower and bee, they also function as advertising signals alongside colour and scent. While the discovery of electroreception in bees changed our way of thinking about pollination in recent years, the mounting evidence of sound perception in plants may well cause similar ripple effects in the near future (Khait *et al*., 2019). That plants can sense gravity has long been known and is evident from gravitropic growth responses (Moulia and Fournier, 2009; Bérut *et al*., 2018; Sierra-de-Grado *et al*., 2022). In this issue, Gallep and Robert (2022) move on to challenge our understanding of the regulation of circadian rhythms by linking them to fluctuations of gravitational forces as a result of the lunisolar tide cycles.

With regard to our initial focus on increasing mechanical stresses as a result of changing climate and weather patterns, the most obvious mechanical demand for plants is damage resistance to the forces of hail, wind (Gardiner *et al*., 2016), and waves (Koehl, 2022). Several articles in this issue explore the response of leaves to mechanical loading and impacts. Mimi Koehl (2022) provides a comprehensive overview of the adaptations and responses of marine macroalgae and seagrasses to the extreme mechanical loads in their wave-swept habitats. Burnett and Gaylord (2022) complement this ecological perspective with a more technical viewpoint on the frameworks, methodologies, and challenges of measuring wave forces and exposure in the field. Moving to terrestrial ecosystems, Langer *et al*. (2022) investigated the detailed responses of petiole, lamina, and the interjacent transition zone of peltate *Pilea piperomioides* leaves to wind loading in unprecedented detail. Interestingly, they found clear differences in the biomechanical properties of each component, but no adaptive plasticity in the form of thigmomorphogenetic acclimations in response to prolonged wind exposure. Lauderbaugh and Holder (2022) and Roth-Nebelsick *et al*. (2022) both apply a biomechanics approach to the detailed characterization of leaf responses to rain drop impacts, while Lenz *et al*. (2022) integrate the biomechanical interaction of leaves with rain into the broader ecological and physiological context. They add to previous calls for performing biomechanical measurements on naturally growing plants in the field (Bauer *et al*., 2020), pointing out the shortcomings of purely correlative field studies on the one hand, and of the extrapolation of lab results to the field on the other hand. Taking biomechanical measurements out into the field is undoubtedly challenging, but modern advances in measurement technology open up unprecedented opportunities. New low-cost instrumentation and fully customizable modular set-ups allow autonomous data collection in the field, often over extended periods of time (Kwok, 2017; Oellermann *et al*., 2021, Preprint; Burnett and Gaylord, 2022). Almost ironically, the pioneers of ‘field biomechanics’ were often marine biologists, despite the sea and the tidal zones being undoubtedly amongst the most challenging environments to which one could take measurement electronics (Bell and Denny, 1994).

If we master the challenges of combining experimental and theoretical approaches from engineering and physics with ecological field experiments, we will not only have a real chance to improve our understanding of the physical demands on plants and their adaptive responses to these demands (Niklas and Telewski, 2022), but we might even be able to tap into plants’ potential to mitigate the effects of climate change and tackle the great challenges of the 21st century. Understanding how plants interact with wind, waves, and precipitation can inform forestry planning and programmes aimed at preventing or limiting erosion and stabilizing coastlines in times of rising sea levels and more frequent storm surges (Denny, 2021). Elucidating the mechanical resilience of plants to damage from abiotic impacts (hail, rain, and wind) or herbivore and pathogen attacks can inform crop breeders of traits to select for, eventually creating more resilient crops that produce higher yields and need fewer pesticides. A more comprehensive understanding of the factors underlying plant–insect interactions can help to conserve pollinator networks and predict challenges for vegetation regeneration due to effects of climate change and anthropogenic disturbance on pollination and seed dispersal.

Seeing the growing scientific interest in mechanical ecology on both sides of the Atlantic and the breadth of research and topics leaves us with a ‘buzz’—an excited anticipation of things to come over the next decade. Mechanical ecology in the sense of field biomechanics truly feels like an interdisciplinary field on the cusp of fully taking off. This Special Issue, along with dedicated conference sessions at the most recent SICB (2022: S10 Integrating ecology and biomechanics to investigate patterns of phenotypic diversity: evolution, development, and functional traits) and upcoming SEB (2022: Mechanical ecology—taking biomechanics to the field) meetings, certainly attests to the timeliness of the topic.
